# Supplementary material for: Dual-functional cerium oxide nanoparticles with antioxidant and DNase I activities to prevent and degrade neutrophil extracellular traps
Source: Front Immunol. 2025 Oct 23;16:1693809. doi: 10.3389/fimmu.2025.1693809 (PMC12589997; doi:10.3389/fimmu.2025.1693809)
Supplement: Supplementary file 1 [file DataSheet1.docx]

**Dual-Functional Cerium Oxide Nanoparticles with Antioxidant and DNase-I activities to prevent and degrade Neutrophil Extracellular Traps**

Hachem DICH^§,1^, Ramy ABOU RJEILY^§,1^, Gabriela RATH^1^, Mathéo BERTHET^2^, Bénédicte DAYDE-CAZALS^2^, Jean-François BERRET^3^, Eduardo ANGLES CANO*^,1^

^1^Faculté de Santé, Université Paris Cité, INSERM, Optimisation thérapeutique en neuropharmacologie, U1144, 75006, Paris, France.

^2^Specific Polymers, ZAC Via Domitia, 150 Avenue des Cocardières, 34160 Castries, France.

^3^Université Paris Cité, CNRS, Matière et Systèmes Complexes, 75013 Paris, France.

^§^ H.D. and R.A.R. contributed equally to this work.

*Email: Eduardo.Angles-Cano@inserm.fr

**SUPPLEMENTAL MATERIAL**

**Supplemental Method-1 Coating protocol**

To investigate the interaction and phase behavior of cerium oxide nanoparticles (CNPs) with phosphonic acid-based functional polymers, we used the method of continuous variation in combination with electrostatic complexation principles (1-3). Both polymer and CNP dispersions were prepared under identical acidic conditions (pH 1.4) and at matched concentrations, here c_O_ = 1 g L-1. They were then mixed across a broad range of volumetric ratios (X), from 10⁻³ to 10³, covering polymer-rich (X≪1) to nanoparticle-rich (X≫1) conditions. A key observation from this approach was the identification of a critical mixing ratio, X_C_, below which the resulting nanoparticle–polymer complexes remained colloidally stable across a broad pH window (1 < pH < 9) (2). In contrast, mixtures with X > X_C_ exhibited aggregation or precipitation. This destabilization is attributed to incomplete surface coverage and weak screening of the van der Waals attractions. The final preparation steps for in vitro use of the CNPs involved filtration through a 0.22 µm cellulose acetate membrane, concentration of the dispersions using a 50 kDa cut-off AMICON filter to achieve a final concentration of 20–50 g L⁻¹, followed by autoclaving at 120 °C and 2 × 10⁵ Pa for 2 hours. The resulting dispersions were stored at 4 °C.


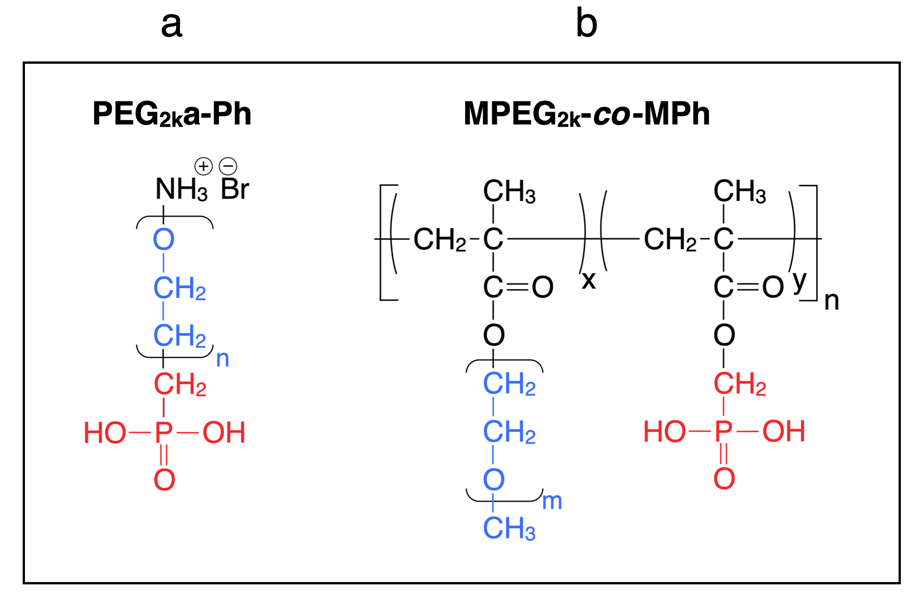


**Supplementary Figure -1**: **Chemical structures and abbreviations of the polymers used in this work** (2). **(a)** Poly(ethylene glycol) with the molecular weight 2 kDa terminated with a single phosphonic acid on one side and with a tertiary amine on the other. The polymer is abbreviated as PEG_2k_a-Ph. **b)** Statistical copolymer MPEG_2k_-*co*-MPh made from MPEG_2k_ and MPh monomers, wherein MPEG_2k_ refers to a PEG methacrylate macromonomer with PEG molecular weight of 2 kDa and MPh to a methacrylate monomer bearing a phosphonic acid functional group. The comonomer proportions are listed in Supplementary Table I.


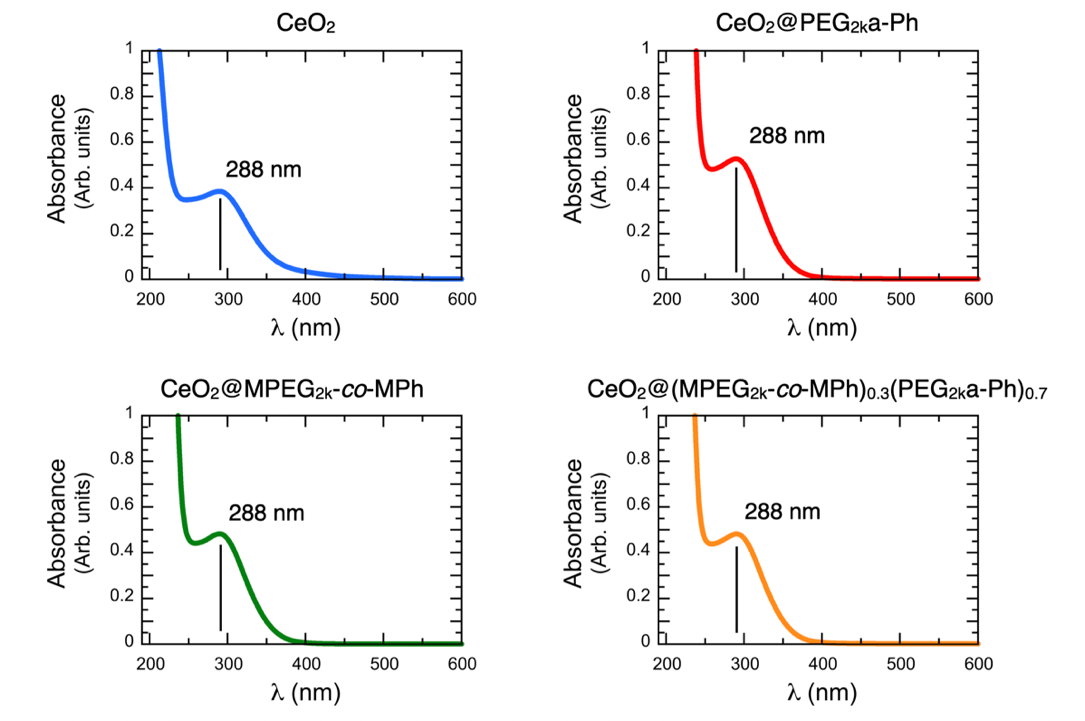


**Supplementary Figure 2. UV-visible spectra of bare and coated nanoparticles.**

Representative absorbance spectra of bare and polymer-coated cerium oxide nanoparticles (CNPs), diluted to concentrations between 2 × 10⁻² and 4 × 10⁻² g L⁻¹, exhibit a characteristic peak at 288 nm. This peak, attributed to cerium oxide, remains unchanged upon coating. According to Beer–Lambert’s law, the absorbance at 288 nm can be used to determine nanoparticle concentration in dispersion.

**Supplemental Method-2 Polymer coating**

The polymers used for coating oxide nanoparticles were synthesized by Specific Polymers via free radical polymerization. They are functional polymers featuring two key components: a phosphonic acid group for anchoring to the positively charged CeO₂ surface, and a polyethylene glycol chain for stability and preventing protein adsorption. For targeting NETs, two different polymers were synthesized: i) one homopolymer terminated by a single acidic phosphonic acid on one side and a primary amine at the PEG end, denoted PEG_2k_a-Ph, and ii) a random copolymer referred to as MPEG_2k_-co-MPh, where MPEG_2k_ denotes a PEG methacrylate monomer with a PEG molecular weight of 2 kDa, and MPh stands for a methacrylate monomer bearing a phosphonic acid functional group. The polymer structures are provided in Supplementary Figure 1. Of note, for statistical copolymers, surface anchoring is strengthened by the presence of multiple phosphonic acid groups per polymer chain (Supplementary Table I). The resulting coating particles are designated CeO_2_@PEG_2k_a-Ph, CeO_2_@MPEG_2k_-co-MPh and CeO_2_@ MPEG_2k_-co-MPEG_2k_ka-co-MPh, respectively.

| Polymers | $\boldsymbol{M}_{\boldsymbol{w}}^{\boldsymbol{Pol}}$  (g mol^-1^) | $\boldsymbol{M}_{\boldsymbol{n}}^{\boldsymbol{Pol}}$  (g mol^-1^) | $\boldsymbol{x}$ – $\boldsymbol{y}$ | Phosphonic  acids/polymer |
| --- | --- | --- | --- | --- |
| PEG_2k_a-Ph | 2000 | 2000 | - | 1.0 |
| MPEG_2k_-*co*-MPh | 62300 | 21750 | x = 0.48 - y = 0.52 | 9.8 |

**Supplementary Table I:** **Molecular characteristics of the phosphonic acid PEGylated polymers and copolymers synthesized in this work**. The chemical structures of the functional polymers along with the definitions of the comonomer proportions $x$ and $y$ are provided in Supplementary Figure 1.

The nomenclature used here is identical to that of the review article on the application of these functional polymers to nanomedicine (2). For simplicity, CNPs coated with the above functional polymers will also be referred to as PEGylated CNPs. Due to the presence of an amine function at the end of the PEG chain, particles coated with PEG2ka-Ph are positively charged. In contrast, CNPs coated with the CeO_2_@MPEG_2k_-*co*-MPh are neutral. Using dynamic light scattering, it was verified that the coating layer thickness was of the order of 5-10 nm, in line with partially stretched PEG chain dimensions (1).

| **Nanoparticles** | $\boldsymbol{D}_{\boldsymbol{H}}$ **(nm)** | $\boldsymbol{h}$ **(nm)** | $\zeta$ **(mV)** | **Polymers per**  **particle** | **PEG**  **density (nm^-2^)** |
| --- | --- | --- | --- | --- | --- |
| Bare CeO_2_ | 9.0 | 0 | +21 ± 1 | 0 | 0 |
| CeO_2_@PEG_2k_a-Ph | 18.1 | 4.5 | +27.7 ± 0.6 | 103 | 0.38 |
| CeO_2_@MPEG_2k_-MPh | 27.2 | 9.1 | +1.4 ± 0.1 | 15 | 0.28 |
| CeO_2_@MPEG_2k_-MPEGa_2k_-MPh | 31.5 | 11.2 | +5.8 ± 0.3 | 26 | 0.62 |

**Supplementary Table II:** Hydrodynamic diameter ($D_{H}$), polymer brush thickness ($h$), zeta potential ($\zeta$) determined for polymer coated nanoparticles. The two last columns are the number of polymers per particle and the PEG density as determined from complementary measurements (1, 4).


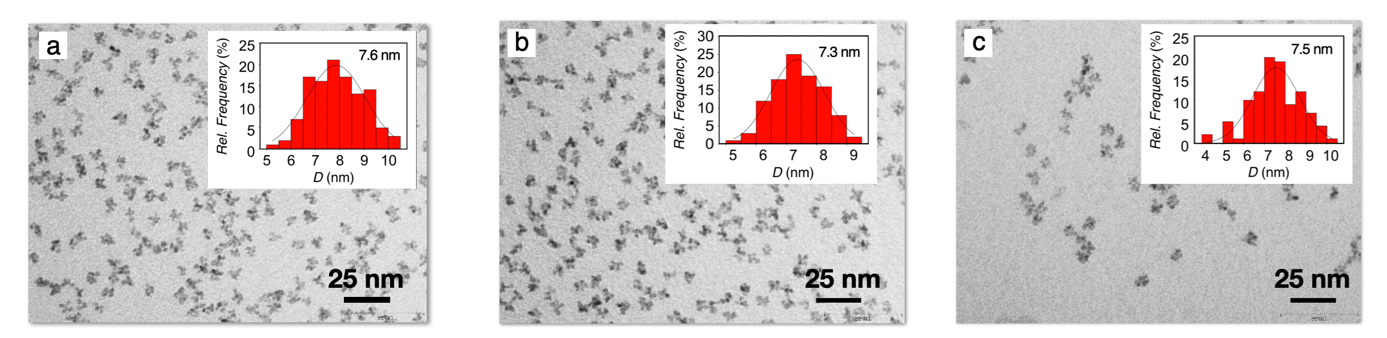


**Supplementary Table III:** Transmission electron microscopy images of cerium oxide nanoparticles: a) CeO_2_, b) MPEG_2k_-co-MPh and c) CeO_2_@MPEG_2k_-MPEGa_2k_-MPh. Insets: size distributions obtained for n = 100 particles. The similarity between the TEM sizes of coated and uncoated particles arises from the very low electron contrast of the polymer shells, which makes them invisible in TEM. However, the presence and thickness of these polymer brushes can be determined by light scattering experiments.

**Supplementary Figure 3 Characterization of NETs in terms of DNA and elastase**
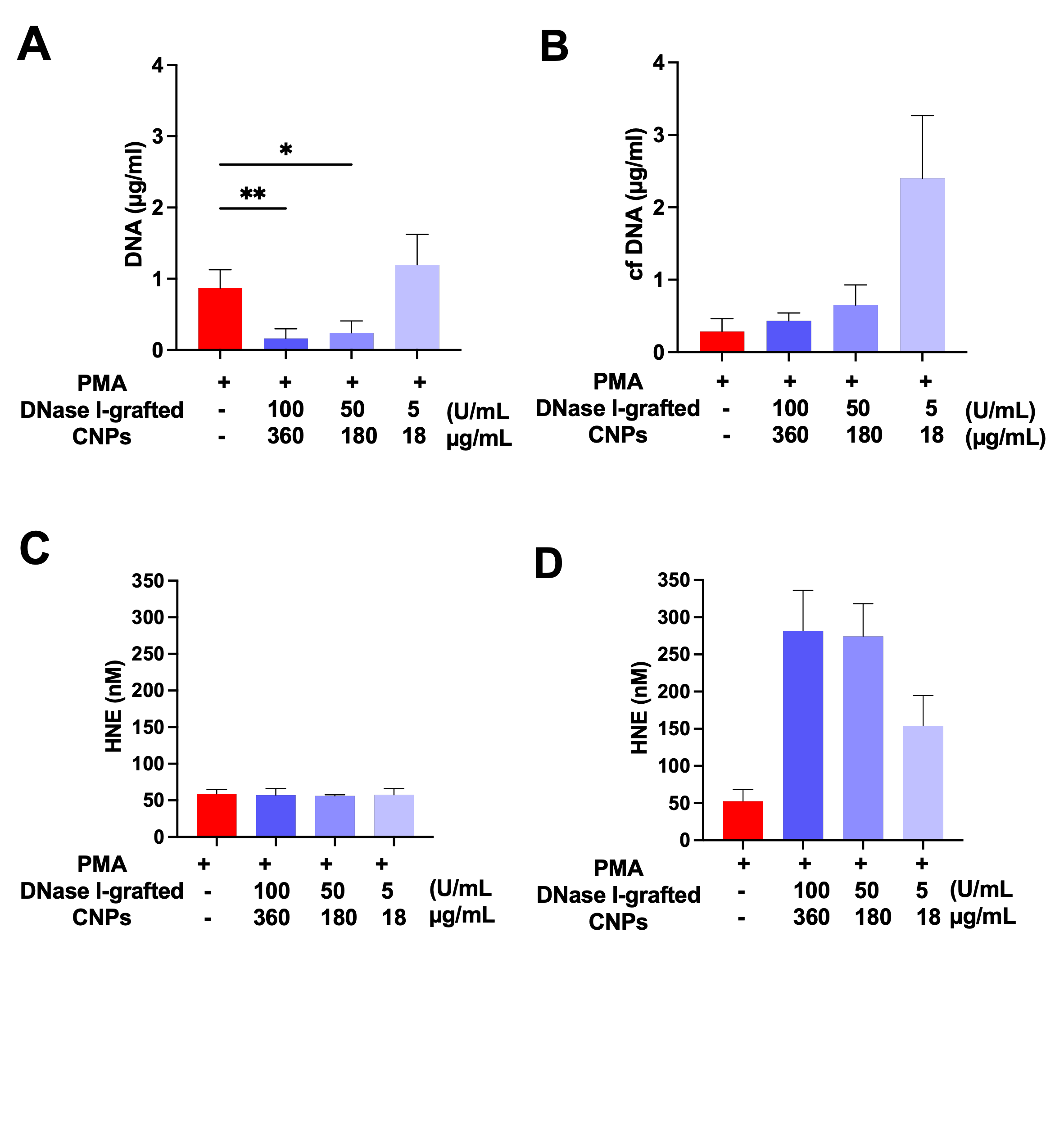


**Supplementary Figure 3 Identification of NETs by quantification of DNA and elastase content.**

Neutrophils were isolated from human blood and seeded onto a 96-well plate at a density of 1 x 10^5^ cells/well. The neutrophils were then incubated with 50 nM PMA (red bars) or buffer alone (HBSS, green bars). The DNase I-grafted nanoparticles were added 30 minutes before PMA stimulation at different concentrations of CNP/DNase I ratio (360 µg/100 U, 180 µg/50 U, and 18 µg/5 U per mL (purple bars from darkest to lightest, respectively). NETs formation was assessed by measuring two key components, DNA using a fluorescent dye (SYBR Green) and human neutrophil elastase (HNE) using a selective chromogenic substrate. Data analysis and graphing were performed with the Prism software. Panels **A** and **B** represent DNA quantification, while panels **C** and **D** show HNE quantification. More specifically the measurements from the wells’ bottom are shown in **A** and **C**, while the measurements from the corresponding supernatants are shown in **B** and **D**. cfDNA: cell-free DNA. Error bars represent SD, n=5, statistical significance is indicated by asterisk *P<0.01, **P<0.004.

**Supplementary Figure-4 Grafting of CY5 onto CNPs (CeO_2_@(MPEG_2k_-co-MPh)₀.₃(PEG_2k_a-Ph)₀.₇ )**

**Cy5 (NHS ester) was used to validate DiOC6 adsorption onto CNPs via electrostatically driven physisorption, chosen to avoid competition with DNase I immobilization.** Supplementary Figure **4 shows intracellular localization of DNA and CY5 (merged images, right panel).**


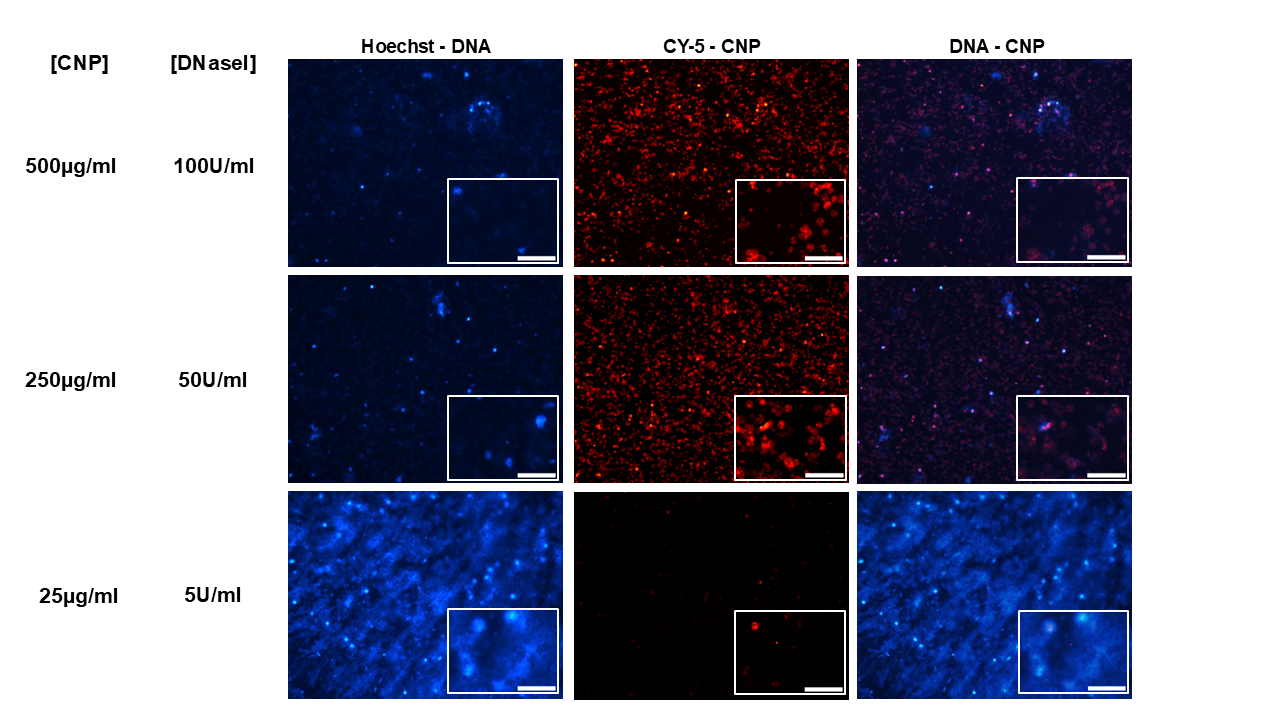


**Supplementary Figure 4 Impact and detection of CeO_2_@(MPEG_2k_-co-MPh)₀.₃(PEG_2k_a-Ph)₀.₇ grafted with DNase-I and CY5.** DNase-I and Cyanine-5 NHS ester (CY5) were immobilized on CNPs as indicated in main text: **Grafting of DNase-I.**

Neutrophils were isolated from human blood and seeded onto a 96-well plate at a density of 1 x 10^5^ cells/well. The cells were then incubated with DNase I-CY5-grafted nanoparticles at various concentrations: (**A**) 100U/mL, (**B**) 50 U/mL, and (**C**) 5 U/mL for 30 minutes. This stimulation was followed by the addition of 50nM PMA to induce the formation of NETs. The CNP/DNase I concentration per mL were 500 µg/100 U, 250 µg/50 U, and 25µg/5 U. The images shown in **A**, **B** & **C** were detected in optic fields of three wells for each condition using the x10 objective of a Zeiss AxioObserver D1 fluorescence microscope equipped with a CCD Imaging camera. n=5. Representative images of fluorescence microscopy using Hoechst 33342 for DNA staining (left column), and CY5 CNP labelling (middle raw). Merged images of the left and middle columns are shown in the right column. Inset (500 % zoom, scale bar 50µM).

**Supplementary Figure-5 CNP antioxidant activity. Coating effect on NETs formation**

We tested CNPs coated with various PEG polymers to identify formulations that retain their antioxidant activity in the presence of neutrophils


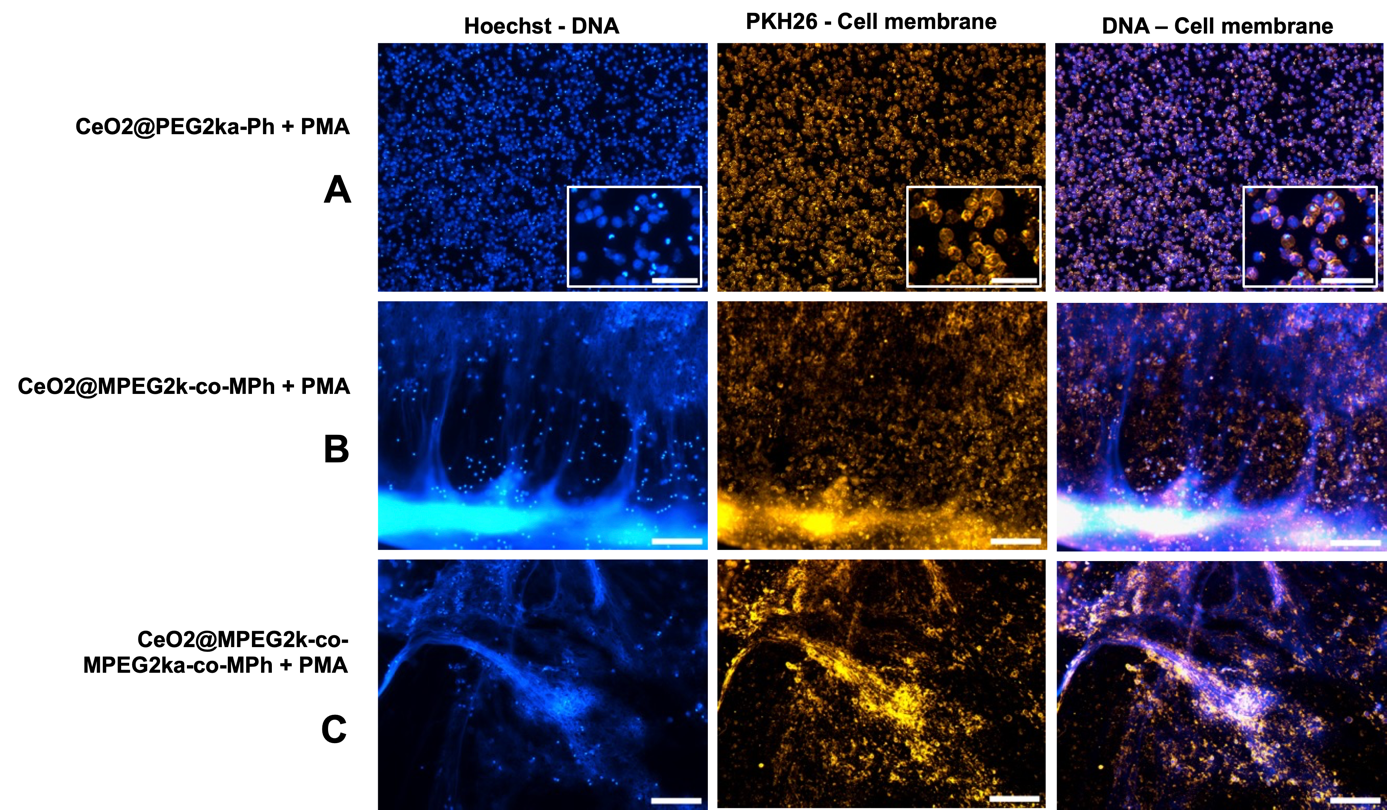


**Supplementary Figure 5. Impact of cerium oxide nanoparticles on PMA-stimulated neutrophils.**

Neutrophils isolated from human blood were seeded on a 96-well plate at 1x10^5^ cells/well as indicated in Figure 1. Nanoparticles coated with different PEGylated polymers: (**A**) CeO_2_@PEG_2k_a-Ph, (**B**) CeO_2_@MPEG_2k_-*co*-MPh, (**C**) CeO_2_@MPEG_2k_-co-MPEG_2ka_-co-MPh, were added at 1000µg/ml to neutrophil-containing wells 30 min prior to stimulation with 50 nM PMA (n=3). Representative images of fluorescence microscopy: Left column: Hoechst 33342 for DNA staining. Middle column: PKH26 staining for membrane labelling. Right column: merged left and middle column images. Scale bar 50 µm (**A** inset) and 150 µm (**B**, **C**). Representative neutrophil fluorescence images captured as indicated in Figure 1.

**REFERENCES**

1. Baldim V, Bia N, Graillot A, Loubat C, Berret J-F. Monophosphonic versus Multiphosphonic Acid Based PEGylated Polymers for Functionalization and Stabilization of Metal (Ce, Fe, Ti, Al) Oxide Nanoparticles in Biological Media. Advanced Materials Interfaces. 2019;6(7):1801814.

2. Berret J-F, Graillot A. Versatile Coating Platform for Metal Oxide Nanoparticles: Applications to Materials and Biological Science. Langmuir. 2022;38(18):5323-38.

3. Torrisi V, Graillot A, Vitorazi L, Crouzet Q, Marletta G, Loubat C, et al. Preventing Corona Effects: Multiphosphonic Acid Poly(ethylene glycol) Copolymers for Stable Stealth Iron Oxide Nanoparticles. Biomacromolecules. 2014;15(8):3171-9.

4. Baldim V, Yadav N, Bia N, Graillot A, Loubat C, Singh S, et al. Polymer-Coated Cerium Oxide Nanoparticles as Oxidoreductase-like Catalysts. ACS Appl Mater Interfaces. 2020;12(37):42056-66.
